# Cryo-EM structure of Hepatitis C virus IRES bound to the human ribosome at 3.9-Å resolution

**DOI:** 10.1038/ncomms8646

**Published:** 2015-07-08

**Authors:** Nick Quade, Daniel Boehringer, Marc Leibundgut, Joop van den Heuvel, Nenad Ban

**Affiliations:** 1 Department of Biology, Institute of Molecular Biology and Biophysics, Otto-Stern-Weg 5, ETH Zürich, Zürich 8093, Switzerland.; 2 Research Group Recombinant Protein Expression, Helmholtz Centre for Infection Research, Inhoffenstraße 7, Braunschweig 38124, Germany.

## Abstract

Hepatitis C virus (HCV), a widespread human pathogen, is dependent on a highly structured 5′-untranslated region of its mRNA, referred to as internal ribosome entry site (IRES), for the translation of all of its proteins. The HCV IRES initiates translation by directly binding to the small ribosomal subunit (40S), circumventing the need for many eukaryotic translation initiation factors required for mRNA scanning. Here we present the cryo-EM structure of the human 40S ribosomal subunit in complex with the HCV IRES at 3.9 Å resolution, determined by focused refinement of an 80S ribosome–HCV IRES complex. The structure reveals the molecular details of the interactions between the IRES and the 40S, showing that expansion segment 7 (ES7) of the 18S rRNA acts as a central anchor point for the HCV IRES. The structural data rationalizes previous biochemical and genetic evidence regarding the initiation mechanism of the HCV and other related IRESs.

## Abstract (web summary)

The Hepatitis C virus (HCV) relies on an internal ribosome entry site (IRES) for translation of all the proteins encoded by its single-stranded RNA genome. Here the authors present a near-atomic cryo-EM structure of the HCV IRES bound to the human ribosome, shedding light on the initiation mechanism of HCV's and related IRESs.

## Graphical abstract

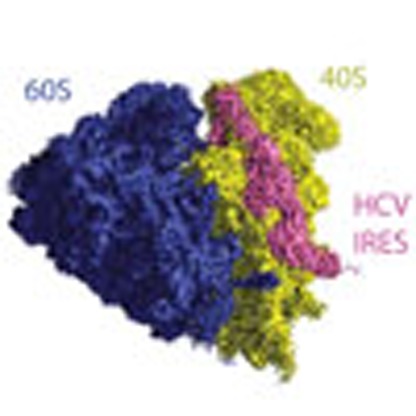

Hepatitis C virus (HCV) is a widespread human pathogen infecting an estimated 150 million people worldwide^1^. HCV virions contain a single-stranded RNA genome and rely on a structured region of its 5′-untranslated region, referred to as the internal ribosome entry site (IRES), for translation of all the encoded proteins. Canonical translation initiation and many IRESs depend on a large number of initiation factors (eIFs) such as eIF1, eIF1A, eIF2, eIF3 and eIF4A and G^2^. In contrast, the HCV IRES requires only eIF2 and eIF3 for start codon recognition, binding directly to the 40S subunit and eIF3 (refs 3, 4). Translation initiation by the HCV IRES begins with binding of the IRES to the 40S ribosomal subunit. The IRES then recruits eIF3 and the ternary complex of eIF2–tRNA_i_^Met^–GTP is bound at the start codon. On GTP hydrolysis eIF2 as well as eIF3 dissociate and the 60S subunit joins to form a translation competent 80S complex with the initiator tRNA positioned at the P-site^5^ (Supplementary Fig. 1).

Due to the central role of the HCV IRES in recruiting host ribosomes for translation of viral mRNA, its architecture and the mode of interactions with the ribosome have been extensively investigated. The HCV IRES as well as several HCV-like IRESs share a common conserved fold encompassing around 300 bases, which form two major domains (II and III)^6^ (Fig. 1a). Electron microscopic (EM) studies have revealed that domain III binds in an elongated conformation at the back of the 40S^7^,^8^,^9^. It is responsible for high-affinity binding and proper positioning of the mRNA in the mRNA channel by establishing direct interactions through subdomains IIIa, c, d, e and f^3^ (Fig. 1b). eIF3 binding has been shown to be mediated by domain IIIb and the junction IIIabc^3^,^9^. Domain II, on the other hand, forms an L-shaped structure that reaches across the 40S subunit and into the intersubunit space^7^,^8^,^10^. It has been demonstrated that domain II induces a conformational change in the 40S^7^ and is involved in release of eIF2 (ref. 11) and transition from translation initiation to elongation^12^.

Although high-resolution X-ray and NMR structures of many fragments of the HCV IRES are available^13^,^14^,^15^,^16^,^17^, interactions of the HCV and CSFV IRESs with the ribosome have only been investigated by cryo-EM at around 8 Å resolution in heterologous complexes with porcine or rabbit ribosomes^9^,^10^. Here we present a 3.9 Å cryo-EM structure of the homologous complex of the HCV IRES bound to the human 40S ribosome, which was solved by focused refinement of the HCV IRES bound to 80S ribosomes. This represents the first near-atomic structure of the HCV IRES that allows visualization of the network of molecular interactions formed between the 40S subunit and the HCV IRES. We identify expansion segment 7, which is tightly bound between two IRES domains, as a central anchor point for the HCV IRES. Furthermore, we show that the HCV IRES and the Cricket paralysis virus (CrPV) IRES, although structurally unrelated, share interactions with ribosomal proteins.

## Results

### Cryo-EM reconstruction of the ribosome-bound HCV IRES

**Figure 1:**
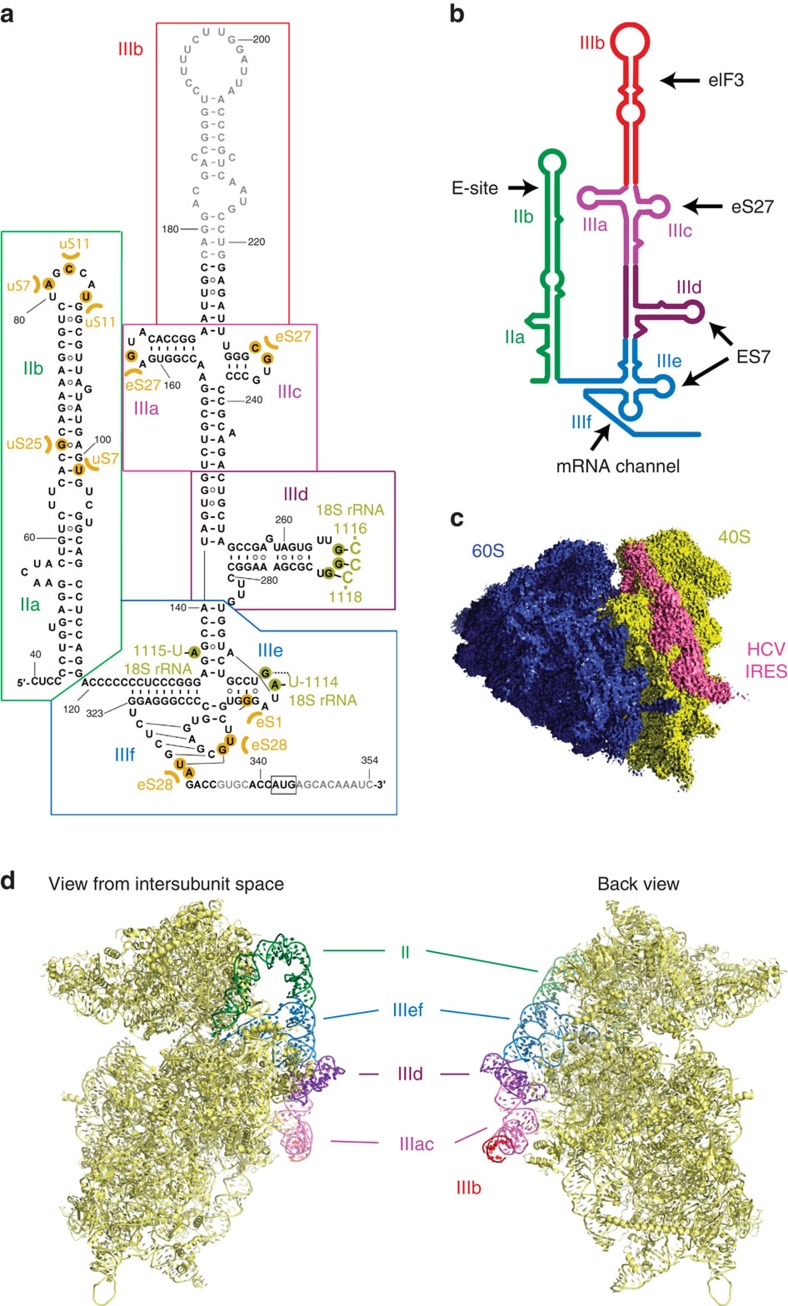
HCV IRES bound to human ribosome. (**a**) Secondary structure diagram of the HCV IRES. The various domains are indicated by different colours and roman numbers. Canonical base pairs are marked by lines and non-standard base pairs by circles. Interactions of the IRES with ribosomal proteins are highlighted in orange, interactions with the 18S rRNA in dark yellow. Bases not built in our structure are indicated in grey, the start codon is indicated by a black box. (**b**) Cartoon representation of the HCV IRES secondary structure. The interaction sites of the different domains are indicated. (**c**) EM density of 80S–HCV IRES complex showing that the HCV IRES does not contact the 60S subunit. The 40S is coloured in yellow, the 60S in blue and the HCV IRES in pink. (**d**) Structure of the HCV IRES bound to the human 40S. Views from the intersubunit side (left) and the solvent side (right).

To investigate the molecular interactions between the HCV IRES and the human ribosome, we reconstituted the 80S–HCV IRES complex from human 80S ribosomes purified from HEK293-6E cells and prefolded HCV IRES. Although our complex contained both ribosomal subunits, initial single particle cryo-EM reconstructions indicated that the IRES in the 80S complex contacts exclusively the 40S subunit and adopts a conformation previously observed in 40S–HCV IRES complexes^7^ (Fig. 1c). The general features of the structure are very similar to a previous structure of the 80S ribosome stalled in initiation by cycloheximide bound to HCV IRES^8^ except that the stalled 80S–HCV IRES complex showed additional density connecting domain IIa of the HCV IRES to the L1 stalk of the 60S subunit. This interaction is not observed in our structure even at low thresholds. The additional density contacting the L1 stalk might be attributable to HCV IRES domain I or the RNA aptamer used for purification located at the 5′ end of the IRES in domain IIa, which were not present in our construct. Formed in the absence of initiation factors or tRNAs, our complex corresponds to the initial binding complex of IRES to the 40S subunit (Supplementary Fig. 1), in agreement with kinetic data that show that binding occurs in a single step and is followed by 80S assembly^18^. Consequently, we focused the alignment on the 40S subunit with bound IRES molecule to obtain a 3.9 Å resolution reconstruction (Supplementary Figs 2,3). Our reconstruction of the human 40S–HCV IRES complex revealed clear density corresponding to the HCV IRES on the back of the 40S. On the basis of structures of individual IRES domains an atomic model could be built for most of the IRES (Fig. 1d, Supplementary Fig. 4), except for a part of domain IIIb (bases 178–221), for which density is weak, apparently due to partial disorder in the absence of eIF3^9^. The single-stranded mRNA region of the IRES was also poorly resolved in our density and was only partially interpreted.

### ES7 acts as a central anchor point for the HCV IRES

**Figure 2:**
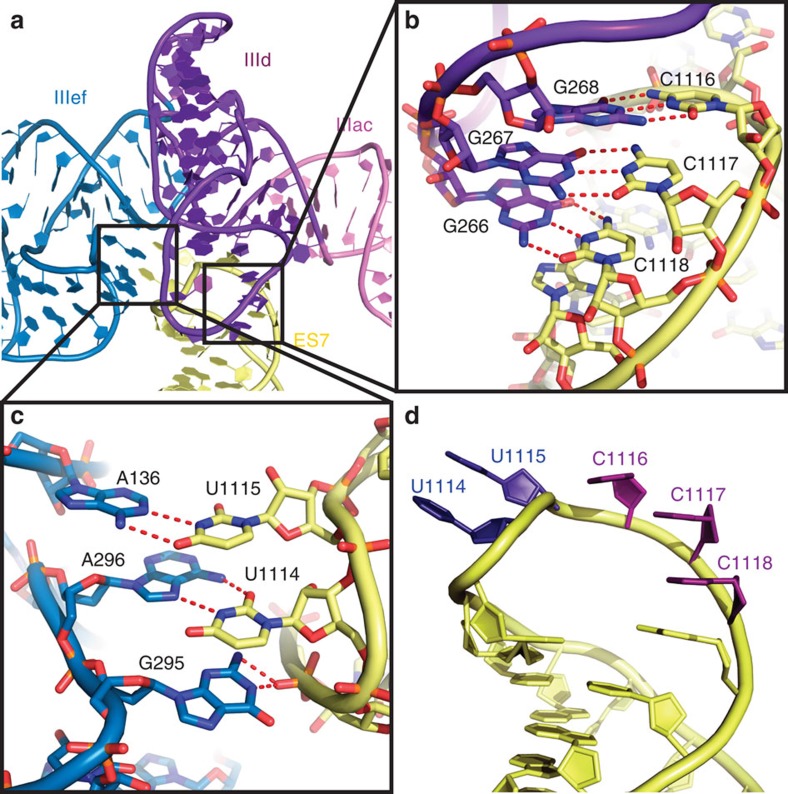
ES7 acts as the main anchoring point for the HCV IRES. (**a**) Overview over the architecture of the binding site. (**b,c**) Close-ups of the interactions between ES7 and domain IIId (**b**) and IIIe (**c**). The structures are shown in cartoon and sticks, red dashes indicate hydrogen bonds. (**d**) Conformation of ES7 bound to HCV IRES. Bases interacting with domain IIId are coloured in purple, and bases interacting with domain IIIef are coloured in dark blue.

The most prominent binding site of the HCV IRES on the 40S subunit is ES7 of the 18S rRNA (bases 1,114–1,118). Specifically, domains IIId and the pseudoknot domain IIIef of the IRES contact two sides of the ES7 stem loop (Fig. 2a). These IRES domains have been identified biochemically to provide most of the binding affinity of the HCV IRES for the 40S subunit^5^ and are critical for the initial binding of the HCV IRES^3^,^5^. A detailed view of this contact area explains the molecular basis of the nanomolar affinity of the HCV IRES for the 40S subunit by revealing an intricate network of interactions between ES7 and domains IIId and IIIe (Fig. 2b,c). The apical loop of domain IIId forms a kissing loop interaction with ES7, which becomes ordered on HCV IRES binding in comparison with the structure of the free porcine 40S subunit, where ES7 exhibits considerable flexibility^19^ (Fig. 2d). The highly conserved^20^ bases ^266^GGG^268^ in the loop of HCV IRES domain IIId form Waston–Crick base pairs with residues ^1116^CCC^1118^ within ES7 (Fig. 2b, Supplementary Fig. 4b). This interaction explains the previous observations that mutations of these guanosine residues lead to reduced IRES activity, and that activity can be rescued by compensatory mutations in ES7(ref. 21). Intriguingly, a stem-loop structure involved in translation termination/reinitiation of caliciviruses containing three guanosine residues has been shown to directly interact with the same bases of ES7 (ref. 22), suggesting that the observed binding mechanism may also be utilized by other RNAs.

On the other face of ES7, bases ^1114^UU^1115^ are flipped out and contact several residues of IRES domain IIIe and a helical region adjacent to it (Fig. 2c,d. Supplementary Fig. 4c–e). In this helical region, HCV IRES base A136 is flipped out and forms a Watson–Crick base pair with residue U1115 of ES7, while A296 and G295 from the loop of IRES domain IIIe interact with U1114 of ES7. A296 forms a reverse Hoogsteen base pair with U1114, and G295 engages in stacking interactions. Bases A136, G295 and A296 are highly conserved among HCV-like IRESs and mutations in G295 and A296 lead to severe defects in translation initiation^23^. The unusual conformation of the IRES at this interaction site is further stabilized by a Watson–Crick base pair between U297 on domain IIIe and A288 in the helical region (Supplementary Fig. 5), which leaves A136 flipped out in the correct position to interact with ES7. The importance of the tertiary interaction between A288 and U297 is supported by the strong conservation and covariation among HCV-like IRESs^20^ and mutational studies showing that single base substitutions can have a significant effect on translation efficiency^13^,^24^. While this domain was visualized previously in a crystal structure^13^, our ribosome-bound structure now reveals how this complex fold enables high-affinity ribosome binding by stabilizing the conformation of A136 and the loop of domain IIIe to provide an interaction site for the flipped out U1114/U1115 of ES7.

In summary, the observed network of specific contacts between the IRES domain IIId and IIIe with ES7 of the 18S rRNA now rationalizes how ES7 can serve as the central anchor point for the HCV IRES and other HCV-like IRESs on the 40S subunit.

### Binding of HCV IRES and eIF3 to eS27 is mutually exclusive

**Figure 3:**
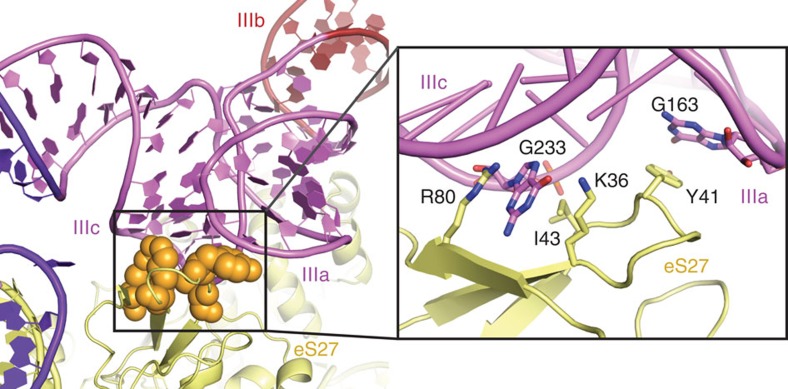
Domain IIIac binding site on eS27. Orange spheres indicate amino acids interacting with the IRES. Inset panel: detailed interactions between bases G163 and G233 of domain IIIac with amino acids from eS27. The structure is shown as cartoon, interacting residues as sticks.

**Figure 4:**
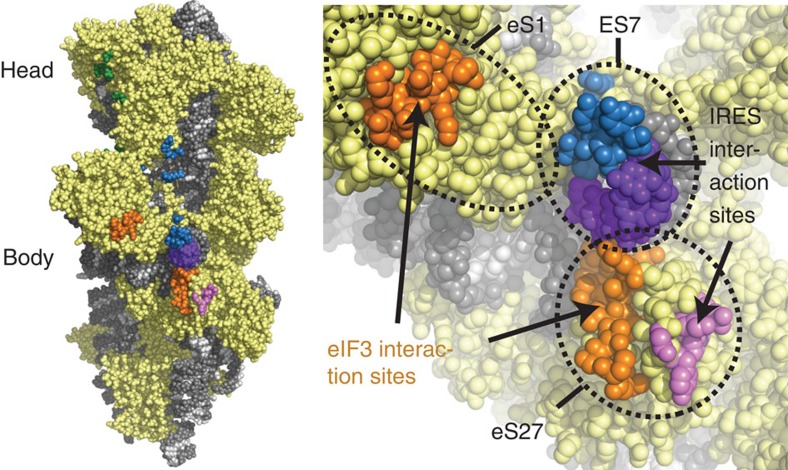
The HCV IRES and eIF3 occupy distinct binding sites on the 40S. Ribosomal proteins are coloured in yellow, the bases of the 18S rRNA are coloured in light grey, the backbone in dark grey. eIF3-binding sites are shown in orange, HCV IRES binding sites are coloured according to their interacting domain (green: domain II, blue: domain IIIef, purple: domain IIId and pink: domain IIIac). The figure was generated by fitting the structure of the 40S bound to the HCV IRES into the density of 40S bound to eIF3 (ref. 26) (EMDB ID 5658).

The second most prominent interface between the HCV IRES and the ribosome involves domains IIIa and IIIc, which contact ribosomal protein eS27 (Fig. 1a, Fig. 3, Supplementary Fig. 4f). The four-way junction between these domains assumes an antiparallel conformation, placing domains IIIa and IIIc close together forming the binding platform for eS27 similar to previous structures^9^,^10^. This is in contrast to the crystal structure of this domain^16^, which assumes a parallel conformation positioning domain IIIb close to the stem connecting the junction to domain IIId and separating domains IIIa and IIIc. It is possible that both conformations exist in solution^25^ and the antiparallel conformation is stabilized on ribosome binding. The binding of domains IIIa and IIIc to eS27 is mainly mediated by the apical loops via two flipped out guanosine residues: G163 in domain IIIa is stacked against the aromatic moiety of Y41, and G233 in domain IIIc forms specific interactions with residues R80, K36 and I43 of eS27. The apical loop of domain IIIa, including base G163 that interacts with the ribosome, is conserved among several HCV-like IRESs, such as CSFV^20^. Interestingly, eS27 is used during canonical initiation as a major contact point for the multisubunit eIF3 complex^26^. In the 11 Å resolution cryo-EM reconstruction of the 40S subunit in complex with eIF3, the initiation factor binds to the opposite face of eS27 compared to the HCV IRES (Fig. 4). Therefore, the binding of the IRES likely excludes eIF3 from binding to the 40S by occupying the same space above the ribosome rather than competing for the identical contact point on the subunit (Supplementary Fig. 6a,b), resulting in displacement of eIF3 as observed in the cryo-EM structure of the 40S in complex with CSFV IRES and eIF3^9^. In this structure, the CSFV IRES adopts a very similar overall conformation as the HCV IRES in our structure, except for the missing domain II in the CSFV construct, the non-homologous extension of domain IIId and domain IIIb, which is mostly disordered in our structure, but ordered in the CSFV structure probably due to the interactions with eIF3.

### Domain IIIf properly positions the start codon

**Figure 5:**
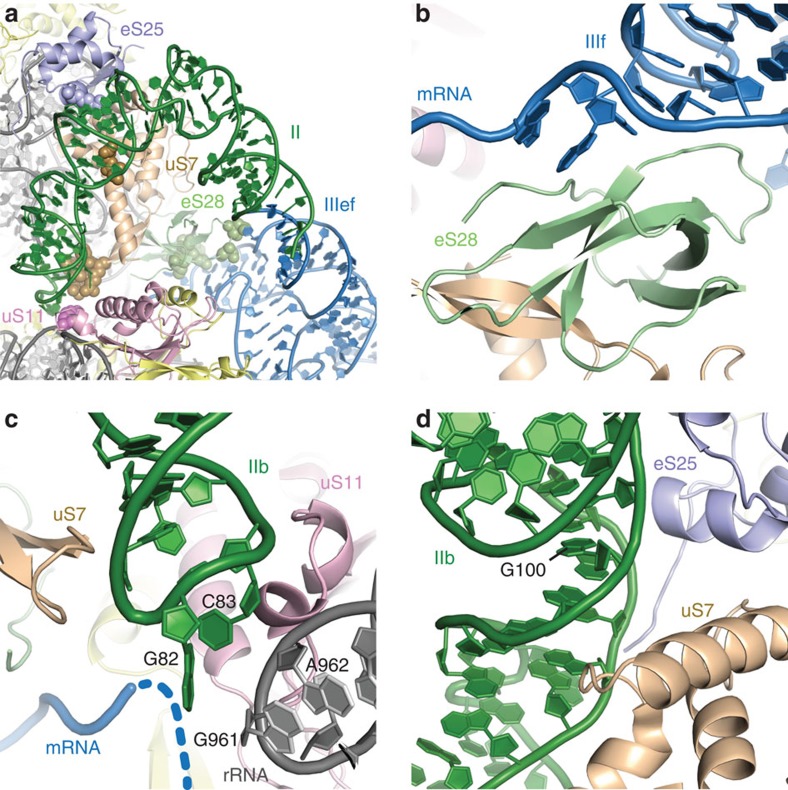
Interactions between ribosomal proteins and domain IIIf and domain II of the HCV IRES. (**a**) Overview over HCV binding sites. Protein uS7 is shown in ochre, uS11 in pink, eS25 in light blue and eS28 in light green. Other ribosomal proteins are coloured in yellow, 18S rRNA in grey and domain II and IIef in green and blue, respectively. Interacting residues are marked by spheres. (**b**–**d**) Detailed views of binding sites of domain IIIf to eS28 (**b**), the apical domain of domain II to uS7 and uS11 (**c**) and a minor interaction site between domain II and uS7 and eS25 (**d**). Structures are depicted as cartoon. The dashed blue line indicates the approximate path of the mRNA.

HCV IRES also interacts extensively with ribosomal protein eS28 at the exit of the mRNA channel via the pseudoknot in domain IIIf (Fig. 5a,b, Supplementary Fig. 4g). The contacts involve hydrophobic interactions with L18 and I43, while the phosphate backbone of the IRES is complemented through charge–charge interactions with side chains of K16 and R31 and R66 (Supplementary Fig. 7a). These rather unspecific interactions are consistent with the function of domain IIIf to insert the viral mRNA into the mRNA channel and to properly position the start codon^3^,^13^,^27^. After switching to the elongation stage of translation, the mRNA exits the mRNA channel probably by displacing domain IIIf from its binding site at the exit of the channel.

### Domain II binds in the E-site above the mRNA channel

**Figure 6:**
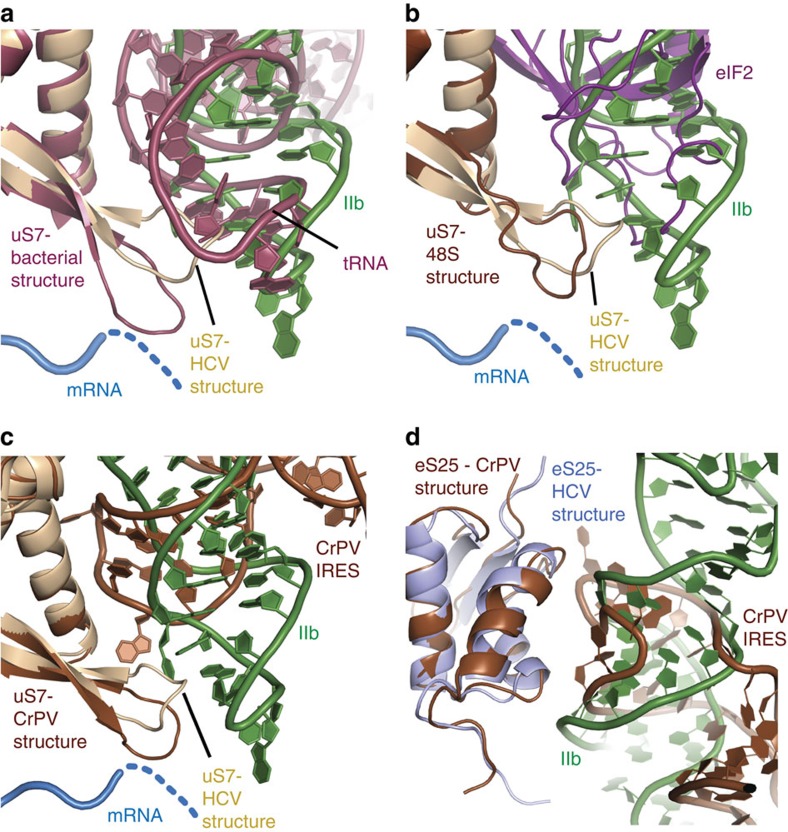
HCV IRES domain II occupies similar binding sites as E-site tRNA, eIF2 and CrPV IRES. (**a**) The apical loop of domain II binds to the β-hairpin of uS7 similar to E-site tRNA. Superposition of the HCV IRES bound to 40S with a bacterial ribosome containing an E-site tRNA^29^ (PDB ID 4RBH) based on uS7. The structure of the bacterial ribosome is shown in red, in the HCV structure domain II is coloured green, mRNA in blue and uS7 in ochre. (**b**) Superposition of the HCV IRES-80S structure and a partial 48S preinitiation complex^32^ (PDB ID 3J81) on uS7 illustrating that eIF2 binding and binding of domain II at the E-site are mutually exclusive. (**c**) Comparison of uS7 interactions of the HCV IRES bound to 80S with the CrPV IRES bound to 80S (coloured in brown). (**d**) Comparison of eS25 interactions of the HCV IRES bound to 80S with CrPV IRES bound to 80S (coloured in brown). The dashed blue line indicates the approximate path of the mRNA.

Domain II is not required for 40S binding^3^ and can adopt a variety of conformations on the ribosome^18^. It has been shown to be important for activation of GTP hydrolysis by eIF2 and subsequent eIF2 release^11^. In addition, domain II is able to manipulate the head–body conformation in the 40S^7^,^10^. In our structure, domain II adopts a bent conformation reaching into the E-site as observed in previous structures^7^,^8^. HCV IRES domain II binds to the intersubunit side of the 40S ribosomal subunit through two spacially well-separated interactions (Fig. 5a). The major binding site is formed by the apical loop of domain II, which reaches into the E-site of the 40S close to the mRNA channel. Here it contacts proteins uS7 and uS11 and is placed in proximity to bases G961 and A962 of the 18S rRNA (Fig. 5c, Supplementary Fig. 4h). The IRES is bound to a β-hairpin structure of uS7 via R130 and R135 as well as T133, and to uS11 by R66 and Y72 (Supplementary Fig. 7b,c). This hairpin structure of uS7 was shown to be involved in contacting both the E-site tRNA and the mRNA codon in the E-site in the structure of a bacterial ribosome^28^. A detailed comparison with an E-site tRNA in the bacterial ribosome^29^ (PDB ID 4RBH) shows that both, domain II of the IRES and the tRNA, bind to the hairpin in a similar fashion using the minor groove of the apical loop of domain II and the anticodon loop of the tRNA, respectively (Fig. 6a). Mutations in the apical loop of domain II, which is conserved in several HCV-like IRESs, including CSFV, have been shown to affect the configuration of the mRNA in the mRNA channel^30^ and severely affect translation efficiency^5^,^31^. It is therefore possible that the placement of domain II in the E-site and interaction with uS7 leads to a 40S conformation that is necessary for proper positioning of the mRNA in the mRNA channel. Furthermore, a recent cryo-EM structure of a partial 48S preinitiation complex places eIF2 in direct contact with uS7 (Fig. 6b)^32^. This position is incompatible with binding of domain II of the HCV IRES in the E-site. Therefore, eIF2–GTP–tRNA_i_ might displace domain II and the competition between domain II and eIF2 may play a role in evicting eIF2–GDP after start codon recognition and progression towards 80S assembly^5^. The conformational flexibility of domain II was also demonstrated by a recent medium-resolution structure of the HCV IRES in complex with the 40S subunit, tRNA and eIF5B^10^. Compared with our structure, the main difference can be observed in the position of domain II, which adopts a more extended conformation in the eIF5B complex, leading to a displacement of the apical loop of domain II of >30 Å (Supplementary Fig. 8).

The second, less-specific contact point of domain II with the 40S subunit involves the minor groove of domain II in the vicinity of base pair C67-G100 and ribosomal proteins uS7 and eS25 (Fig. 5d, Supplementary Fig. 7d). Interestingly, even though eS25 is not essential for canonical translation initiation, eS25 was demonstrated to be very important for translation initiation of HCV and several other IRESs such as the CrPV IRES^33^. Comparison of the CrPV IRES in complex with the 80S^34^ with the HCV IRES structure reveals that in spite of very different RNA folds and modes of interaction with the ribosome, both IRESs form similar contacts with the N-terminal helix–turn–helix motif of eS25 as well as the β-hairpin structure and C-terminal region of uS7 (Fig. 6c,d). Therefore, uS7 and eS25 may represent common interaction sites for IRESs necessary for cap-independent translation initiation.

## Discussion

The results presented here reveal the molecular basis of HCV IRES binding to the human 40S subunit to initiate translation of its viral genome in infected cells. The main interaction site between the HCV IRES and the ribosome is located at the tip of ES7 of the 18S rRNA. Here the apical loop of domain IIId and the loop of domain IIIe sandwich the tip of ES7 providing an extensive binding interface. In agreement with biochemical and genetic evidence that suggest an important role for this contact, these interactions probably serve as the main anchor point for the IRES on the 40S ribosomal subunit (Fig. 1a,b). The binding of domain IIIac to protein eS27 orients the IRES, prevents binding of eIF3 to the 40S, and positions domain IIIb for eIF3 binding. The interaction of domain IIIf with protein eS28 attaches the IRES at the mRNA channel such that the start codon of the mRNA is properly positioned in the P-site. Finally, the structure reveals two contact areas between the flexible domain II of the IRES and the 40S, the first one comprising eS25 protein, which plays a role in translation initiation by several other IRESs, and the second one between the apical loop of domain II and the E-site of the 40S subunit, where the E-site tRNA would reside during the elongation stage of translation.

Hepatitis C is a very widespread disease, for which no satisfactory treatment has been developed so far. Several studies have attempted to inhibit translation of the HCV genome with an RNA structure that competes with the IRES for 40S binding^35^ or small molecules that bind to and conformationally arrest the HCV IRES^36^. The detailed structural information of the interactions between the IRES and the ribosome presented here will help in further development of anti-HCV drugs and provide fundamental insights in the mechanisms of IRES initiation.

## Methods

### Cultivation of HEK293-6E cells

The HEK293-6E cell line^37^ was grown as suspension culture in batch and subsequent perfusion mode in an autoclavable stirred 6-l bioreactor. The bioreactor was equipped with a double-membrane stirrer using Accurel S6/2 tubing (Membrana, Wuppertal, Germany) for aeration as well as for perfusion with internal cell retention using the same membrane in hydrophilized mode^38^. Cells were cultivated at 37 °C using 20-l FreeStyle F17 medium (Life technologies) supplemented with 7.5 mM Glutamine. Aeration was controlled at a dissolved oxygen concentration of 40 and 5% CO_2_. Cells were grown until a cell density of 2.5 × 10^7^ cells per ml. The biomass of 6 l culture was collected using a Sorvall RC12BP centrifuge for 30 min at 1,000 r.p.m. The pellets were rinsed with standard PBS buffer, fractionated and transferred to 50-ml Falcon tubes. All steps from collecting were performed at 4 °C. After centrifugation in a Megafuge 40R for 30 min at 1,000 r.p.m. Pellets (15-g wet cells weight per tube) were shock frozen in liquid nitrogen and stored at −80 °C until further use.

### Sample preparation and data acquisition

Human ribosomes were prepared similar to previously described methods^39^. In brief, frozen HEK-293 cells were thawed in threefold excess of lysis buffer (50 mM HEPES pH 7.6, 0.5% NP40, 6 mM MgCl_2_, 300 mM NaCl, 50 nM E-64, 20 μM leupeptin, 20 μM bestatin, 5 μM pepstatin A, 20 μM phenanthrolin, 1 μM PMSF and 2 mM DTT) and incubated on ice for 30 min. The lysate was cleared for 10 min at 12,000 *g* and the supernatant loaded onto a 60% (w/v) sucrose cushion containing 50 mM HEPES pH 7.6, 50 mM KCl, 10 mM MgCl_2_ and 5 mM EDTA. Ribosomes were pelleted by centrifugation at 50,000 r.p.m. in a Ti70 rotor (Beckman Coulter) for 20 h. The supernatant was discarded and the pellet resuspended in 1 ml resuspension buffer (50 mM HEPES pH 7.6, 6 mM MgCl_2_, 300 mM KCl, 1 mM DTT, 6.8% sucrose). The resuspended pellet was centrifuged again for 20 min at 20,000 *g* and the supernatant treated with 1 mM puromycin for 30 min on ice. The ribosomal sample was then loaded onto a 10–40% (w/v) sucrose gradient in 50 mM HEPES pH 7.6, 2 mM MgCl_2_, 150 mM KCl and 1 mM DTT and centrifuged at 24,000 r.p.m. for 17 h in a SW32 rotor. The gradient was fractionated and fractions containing 80S ribosomes were pooled and concentrated as well as buffer exchanged into 80S buffer (20 mM HEPES pH 7.6, 100 mM KCl, 5 mM MgCl_2_) using 100,000 kDa MWCO tabletop-centrifuge concentrators (Sartorius).

HCV IRES was produced by *in vitro* transcription of a linearized plasmid containing the HCV IRES sequence, followed by denaturing polyacrylamide electrophoresis and buffer exchange into water using tabletop-centrifuge concentrators. The IRES was then diluted to 1 mg ml^−1^ in folding buffer (20 mM HEPES pH 7.6, 100 mM K-acetate, 2.5 mM MgCl_2_, 0.25 mM spermidine) and folded by heating to 95 °C for one minute and cooling on ice.

The 80S–HCV IRES complex was assembled by adding 300 nM HCV IRES to 100 nM 80S in 80S buffer and incubation for 10 min at 37 °C. The sample was then applied to Quantifoil R2/2 holey carbon grids (Quantifoil Micro Tools) coated with a thin carbon film (prepared using a Balzers BAE 120 thin-film coating system). Immediately, the grids were blotted with damp filter paper from the front and manually plunged into a 1:2 mixture of ethane and propane at liquid nitrogen temperature^40^. Data collection was performed using a Titan Krios cryo-transmission electron microscope (FEI Company) operated at 300 kV equipped with a Falcon II direct electron detector (FEI Company) at a magnification of 100,719 × (accuracy of magnification calibration approximately 0.5%) and defocus values of −1.5 to −3.4 μm. For automated data collection the EPU software (FEI Company) was used. Images were acquired in movie mode using seven movie frames (combined dose 20 electrons per Å^2^) per exposure (combined exposure time 0.77 s) after discarding the first frame (55 ms). Subequently, the movie frames were aligned using the DOSEFGPU DRIFTCORR software^41^ to correct for beam-induced specimen motion.

### Structure calculation

To discard poor regions on the grid, overview images of the holes were inspected. CTF parameters of the images were estimated with CTFFIND^42^ from within RELION^43^ and the resulting power spectra analysed to discard poor quality micrographs. A total of 638,532 particles was selected with Batchboxer (EMAN 1.9)^44^. Using RELION, an initial round of two-dimensional classification was performed with 100 classes at a pixel size of 5.6 Å on the object scale (80-pixel frames). Classes containing 80S ribosomes were selected and subjected to three-dimensional classification with 10 classes at the same pixel size using a low-pass filtered reconstruction of the 80S ribosome as an initial reference (Supplementary Fig. 1). Again, only homogeneous 80S classes were selected, resulting in a dataset of 404.357 particles. A test-refinement without a mask using only 44,801 particles in RELION yielded a 4.2 Å reconstruction of the 80S–HCV IRES complex, however, the resolution of the 40S and the HCV IRES was significantly lower as the alignment of the particles is dominated by the 60S subunit. Therefore, the full data set was refined using focused alignment in RELION by applying a mask around the 40S and the HCV IRES, as described previously^34^. A 3.9 Å resolution (according to a gold standard refinement procedure^45^) reconstruction of the 40S ribosomal subunit was obtained with clear density for the HCV IRES.

### Model building, refinement and validation

Model building was performed in Coot^46^ and O^47^ using the structure of the pig 40S ribosomal subunit (pdb code 4W23)^19^ as the starting model. As the head and body were in a different conformation in the pig apo-40S ribosomal subunit, they were individually rigid body fitted into the density using Chimera^48^. The sequences of the rRNA and the proteins were mutated to the human sequence. A rearrangement was observed in the structure of expansion segment 7 (ES7), which was completely rebuilt, and many obvious errors in both the 18S rRNA and the ribosomal proteins were corrected using the 3.0 Å yeast^49^ and the 3.7 Å *Tetrahymena thermophila*^50^ structures as a guide. While the appearance of bulky side chains in the EM density allowed the correct assignment of the protein register also in areas of lower local resolution, the exact side chain conformations could not be established for all amino acids with certainty in these regions. As a starting point for building the HCV IRES, the previously published model (4UJC)^10^ was used as well as the crystal or NMR structures of individual domains (domain II (1P5P)^14^, domain IIIc (1IDV)^17^, domain IIIabc (1KH6)^16^, domain IIId (1F84)^15^ and IIIef (3T4B)^13^).

The resulting model was refined against back-calculated structure factors using PHENIX, applying the mlhl target function as described^51^,^52^. Briefly, after one round of rigid body refinement using individual proteins, 18S rRNA segments and the HCV IRES as groups, the structure was refined to convergence using nine cycles of individual coordinate and grouped B-factor refinement (two groups per residue). To preserve proper geometry also in areas of lower local resolution, automatically detected Ramachandran, secondary structure and base-pair restraints were applied throughout. To prevent over-refinement, an appropriate weighting of the geometry versus the crystallographic term was established empirically, aiming at good model geometry with a low *R* value (Supplementary Table S1). To validate the structure, the final model was refined against the two half-set maps using a similar procedure after resetting the B-factors and applying a random coordinate shift of 0.5 Å (Supplementary Fig. 2d).

### Creation of figures

Figures of molecular structures were created using the UCSF Chimera package from the Computer Graphics Laboratory, University of California, San Francisco (supported by NIH P41 RR-01081)^48^ and PyMOL (The PyMOL Molecular Graphics System, Version 1.7 Schrödinger, LLC.). Local resolution plots were generated in ResMap^53^.

## Additional information

**Accession codes:** The cryo-EM map of the 40S–HCV IRES complex has been deposited in the EMDB with the accession code EMD-3019 and the structure of the 40S–HCV IRES complex has been deposited in the PDB with the accession code 5A2Q.

**How to cite this article:** Quade, N. *et al*. Cryo-EM structure of Hepatitis C virus IRES bound to the human ribosome at 3.9-Å resolution. *Nat. Commun.* 6:7646 doi: 10.1038/ncomms8646 (2015).

## Supplementary Material

Supplementary Information Supplementary Figures 1-8, Supplementary Table 1 and Supplementary References
