# Supplementary material for: Cryo-EM structure of Hepatitis C virus IRES bound to the human ribosome at 3.9-Å resolution
Source: Nat Commun. 2015 Jul 8;6:7646. doi: 10.1038/ncomms8646 (PMC4510694; doi:10.1038/ncomms8646)
Supplement: Supplementary Information — Supplementary Figures 1-8, Supplementary Table 1 and Supplementary References [file ncomms8646-s1.pdf]

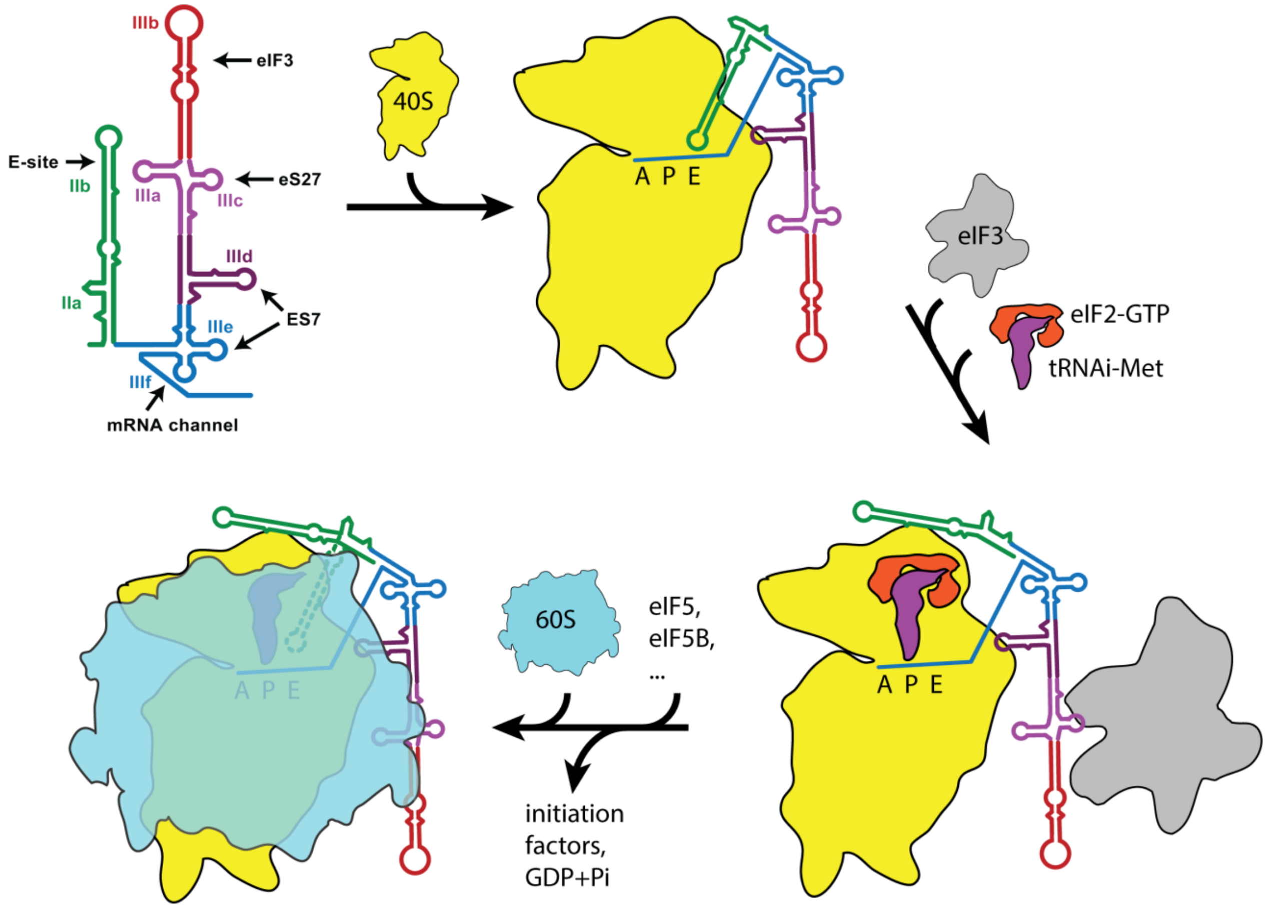


**Supplementary Figure 1** Pathway of translation initiation by HCV IRES. The HCV IRES first binds to the 40S subunit *via* domains IIIac, IIId and IIIef, while domain II resides in the E-site of the 40S. The HCV IRES then recruits eIF3 through domains IIIac and IIIb, and the ternary complex consisting of eIF2, tRNAi$^{Met}$ and GTP binds at the start codon. This probably displaces domain II from the E-site. Upon GTP hydrolysis of eIF2, which is activated by eIF5, eIF2 and eIF3 are released and the 60S subunit joins, assisted by eIF5B, to form a translation competent 80S complex.

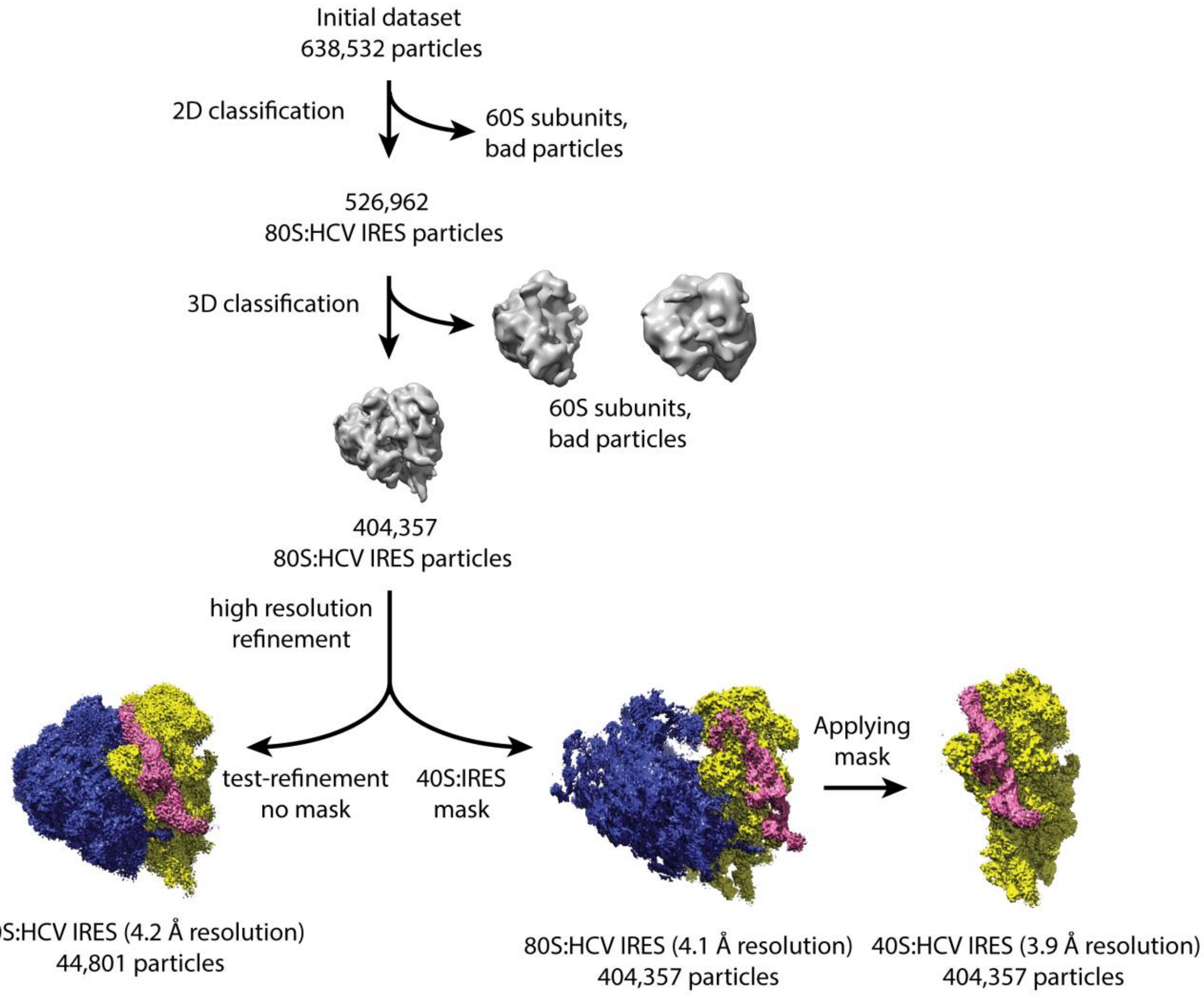


**Supplementary Figure 2** Computational sorting of HCV IRES-40S complexes. In order to obtain a homogeneous dataset for high resolution reconstruction particles were subjected to 2D and 3D classification in RELION[34].

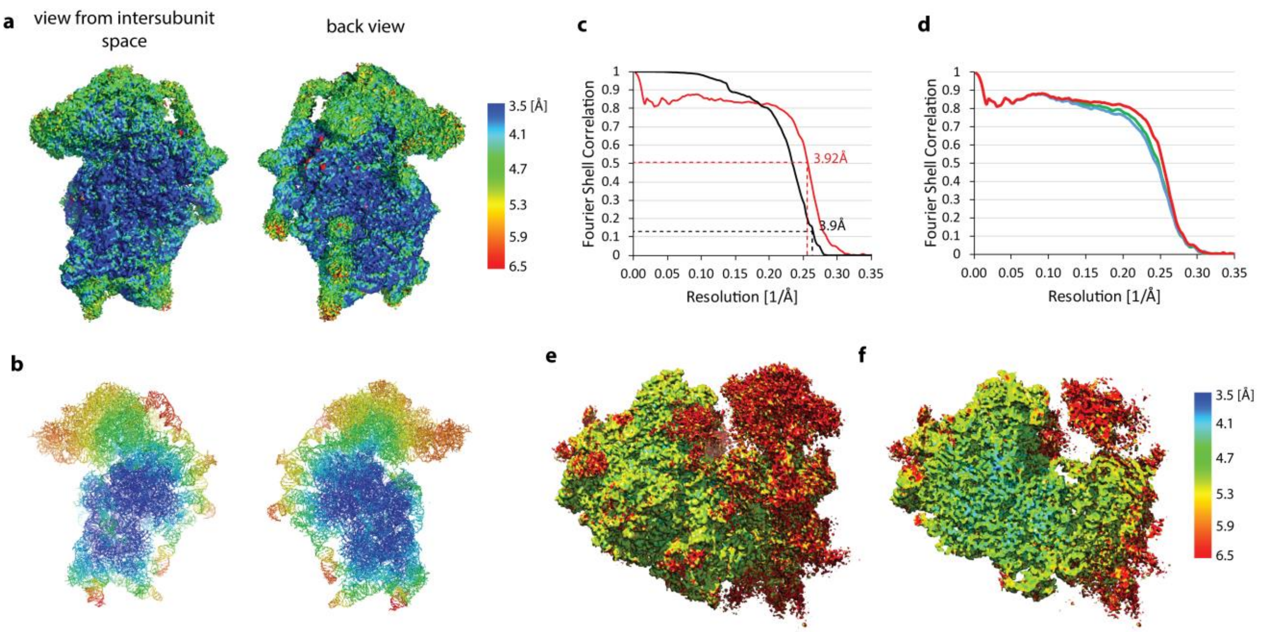


**Supplementary Figure 3** Quality of the cryo-EM map. **(a)** Local resolution plot of HCV IRES bound to 40S. The overall resolution for IRES is about 4Å and at the contact points with the ribosome the resolution is mostly below 4Å. **(b)** The overall B factor distribution of the refined model resembling the local resolution plot of the map (B factors range from 5 to 359). **(c)** Black curve: FSC curve of the 40S-IRES reconstruction. The resolution is 3.9 Å based on gold standard using the FSC = 0.143 criterion. Red curve: FSC curve computed between the final reconstruction and the refined coordinates indicates a resolution of 3.92 Å according to the FSC = 0.5 criterion **(d)** FSC curves calculated for the atomic coordinates refined into maps from one half of the data using a weight of 1.0 for the reciprocal space data (red, FSC model versus map of the full data set; blue, FSC model versus half-set 1 (used for test refinement); green, model versus half-set 2 (not used for test refinement)). **(e,f)** Local resolution plot of test-reconstruction of 80S-HCV IRES complex. Because the alignment is dominated by the 60S subunit, the 40S and HCV IRES have significantly higher local resolution. Therefore, focused refinement on the 40S was performed to obtain high resolution for the 40S and IRES. **(f)** Slice through EM density showing higher resolution at the core of the 80S.

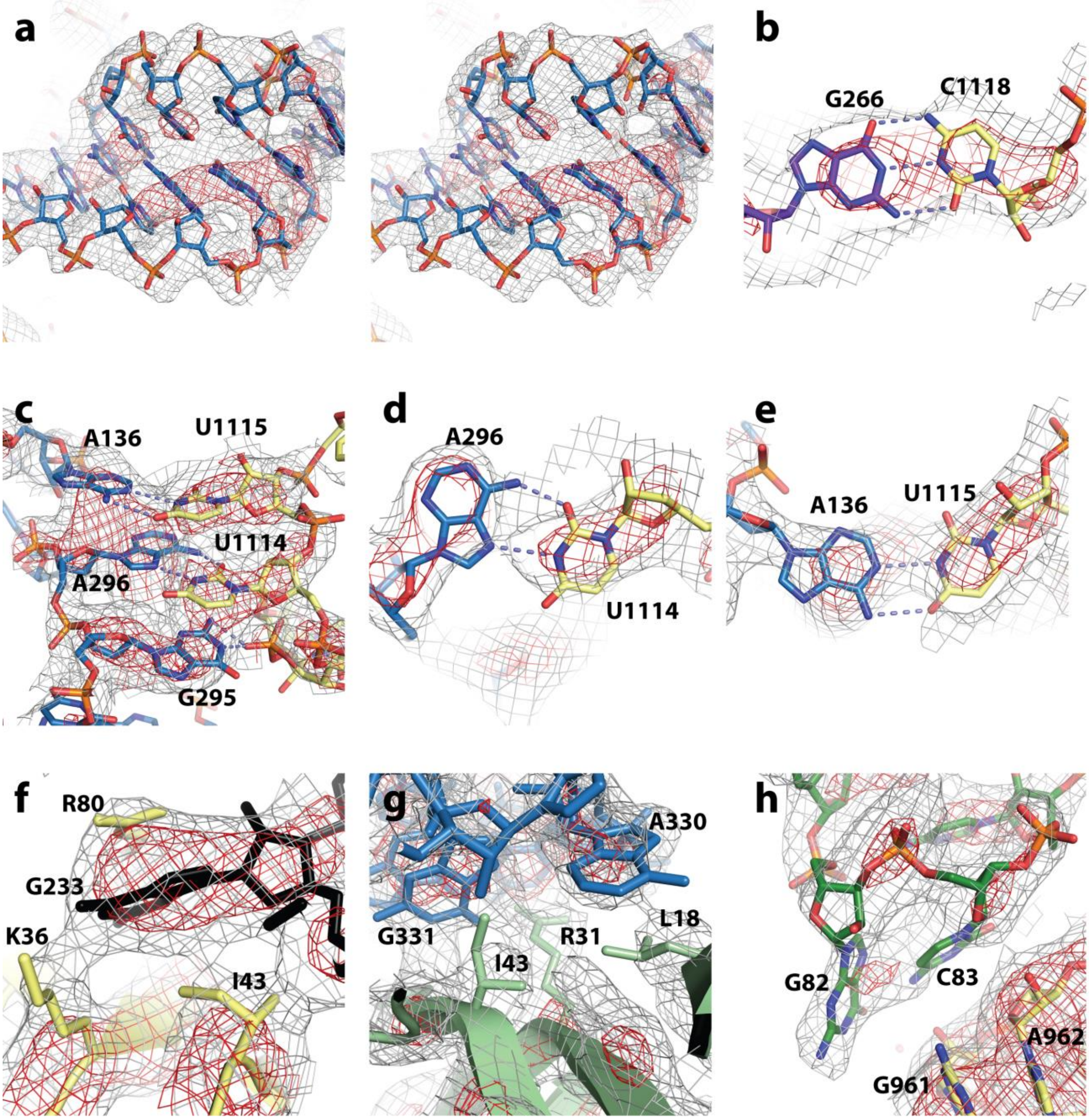


**Supplementary Figure 4** Examples for EM density quality at the ribosome binding sites of IRES. **(a)** Stereo view of density for domain IIIe. **(b)** Top-down view of base pair interactions between ES7 and domain IIId. **(c)** Interaction site between domain IIIef and ES7. **(d,e)** Close-up top-down views on base pair interactions between domain IIIef and ES7. **(f)** Interactions between eS27 (yellow) and domain IIIac (black). **(g)** Interactions between eS28 (green) and domain IIIef (blue). **(h)** EM density for the apical loop of domain II (green) and neighboring ribosomal RNA (yellow).The density is shown at two thresholds (grey: 3 rmsd, red: 7 rmsd).

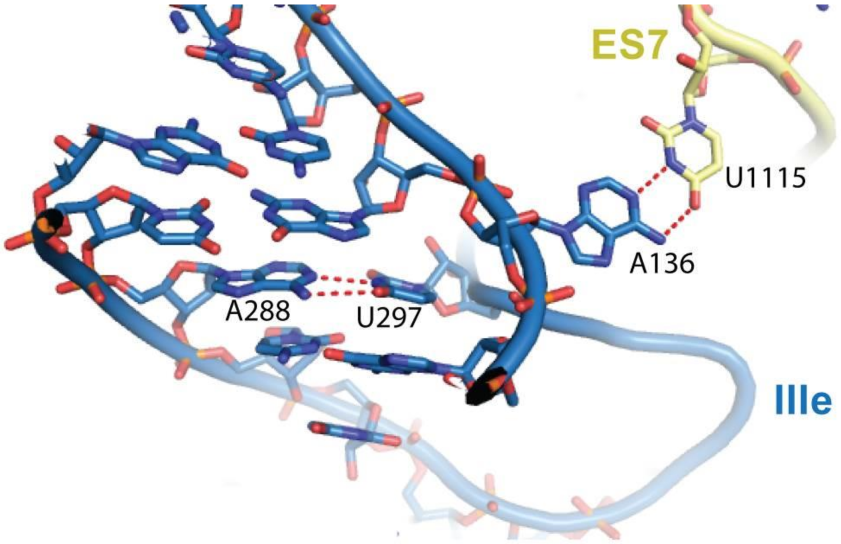


**Supplementary Figure 5** Tertiary interaction between domain IIIe and the adjacent helical region. Structures are shown as cartoon and sticks, hydrogen bonds are indicated by red dotted lines.

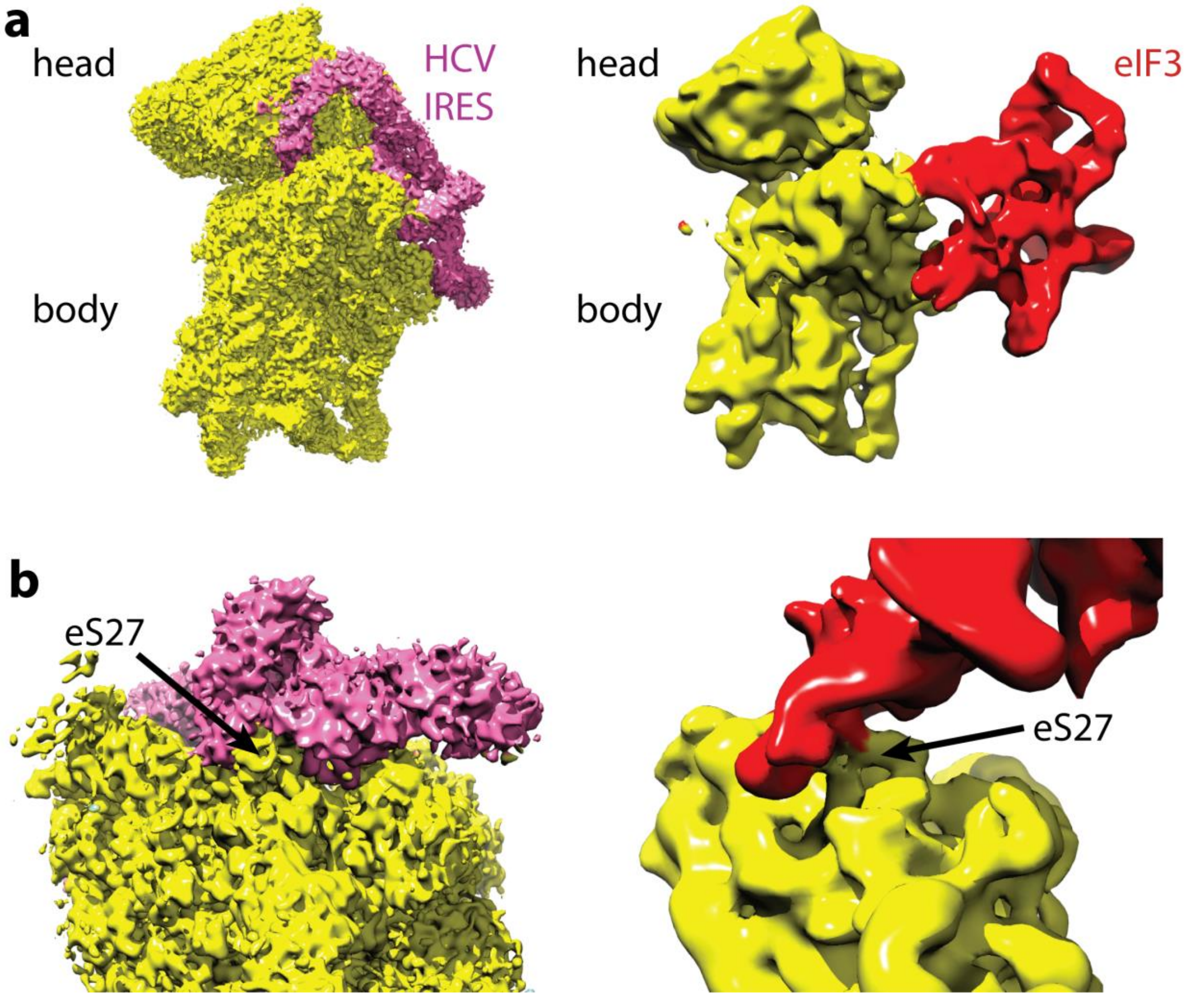


**Supplementary Figure 6** Mutually exclusive binding of the 40S subunit to HCV IRES and eIF3. The EM density of HCV-40S structure and eIF3 bound to 40S (EMDB ID 5658)[1] is shown as a surface representation in yellow for the 40S, pink for the HCV IRES and red for eIF3. **(a)** Side view onto the platform of the 40S. **(b)** Close-up view from the feet of the 40S onto eS27.

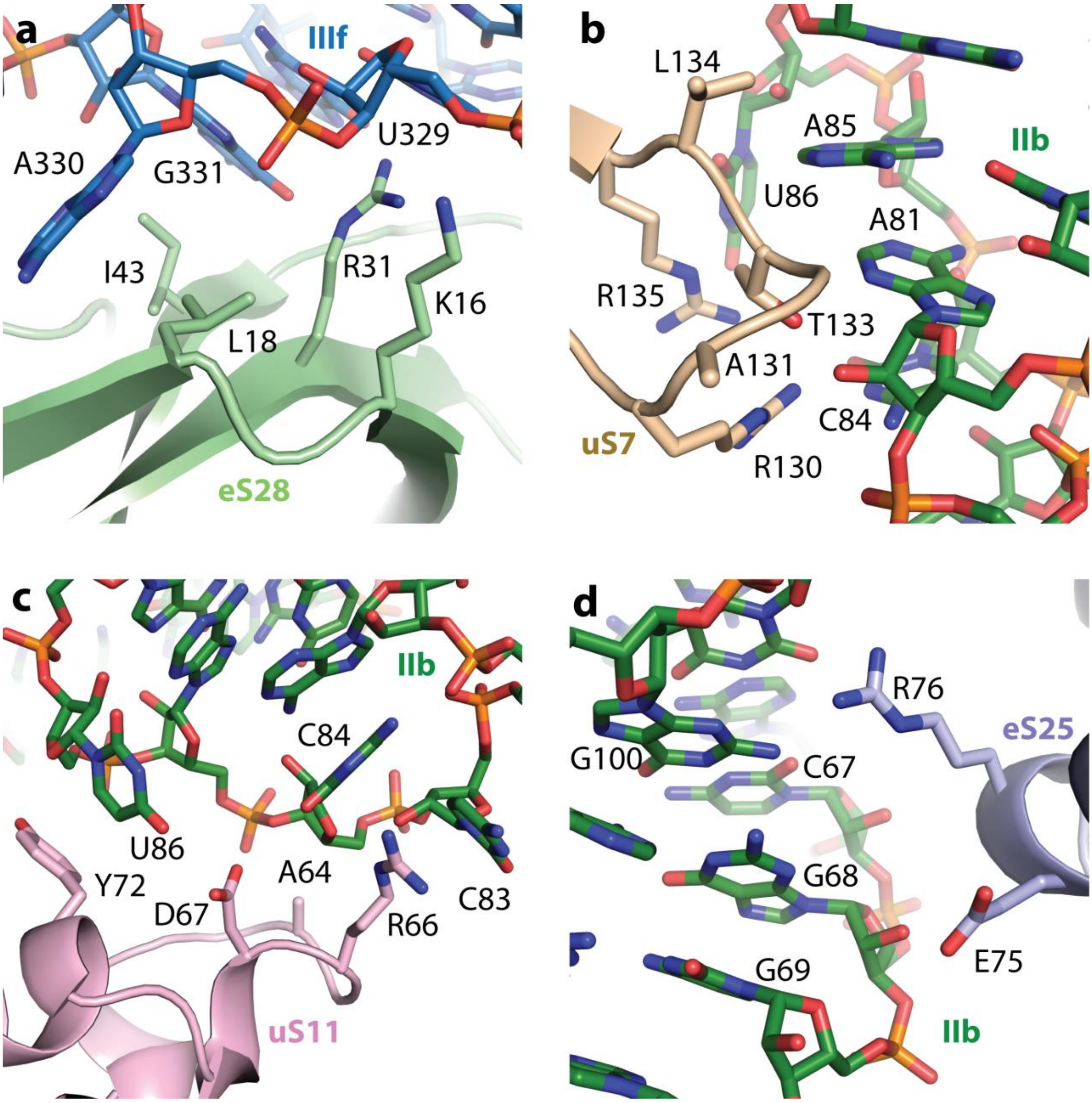


**Supplementary Figure 7** Detailed views of interactions between the HCV IRES and 40S proteins. **(a)** eS28 interacts with the HCV IRES mainly *via* hydrophobic interactions and charge-charge interactions. **(b,c)** The apical loop of domain II interacts with a β-hairpin structure of uS7 **(b)** and uS11 **(c)**. **(d)** eS25 contacts domain II mainly with E75 and R76.

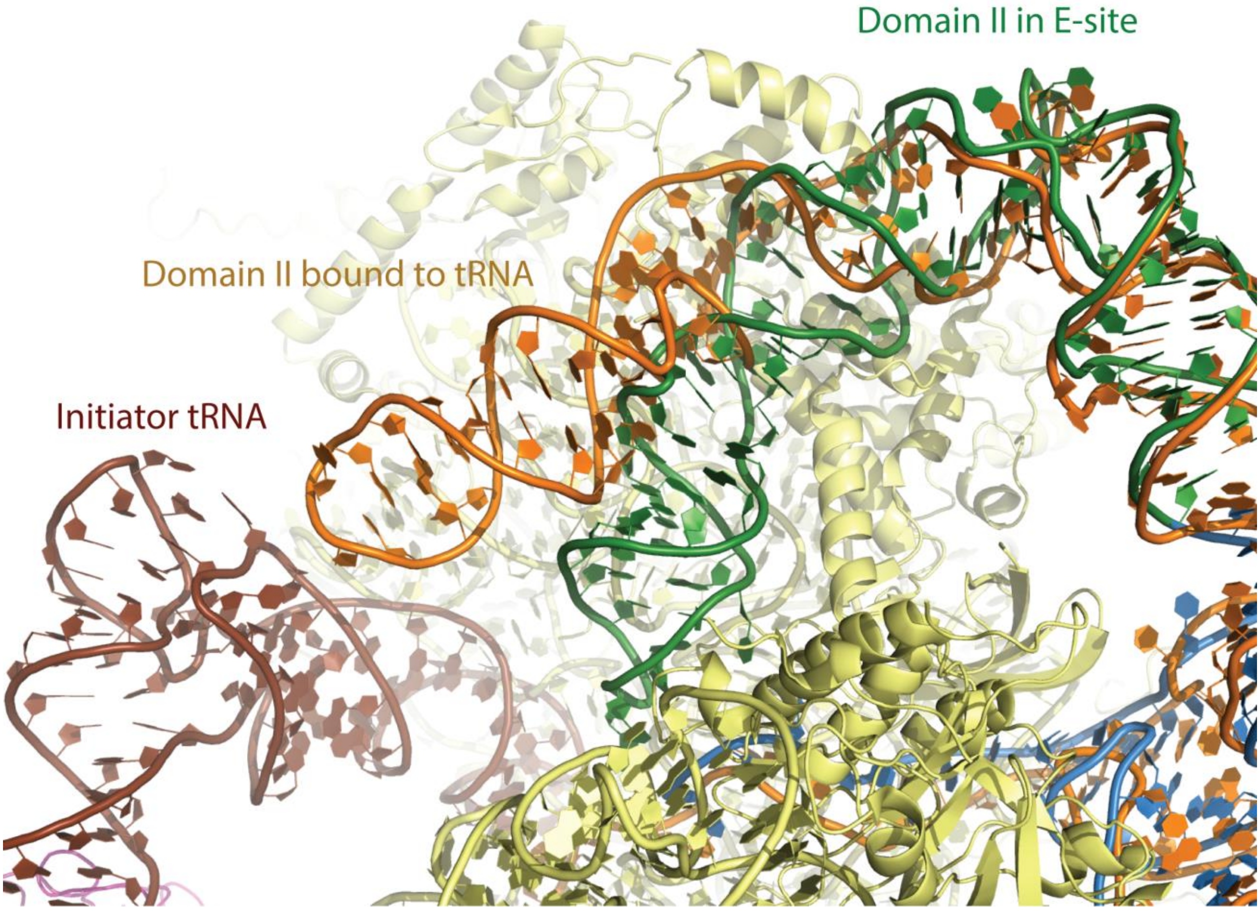


**Supplementary Figure 8** Conformational flexibility of domain II. The HCV IRES-40S structure was superimposed with the structure of HCV IRES bound to 40S, eIF5B and tRNA[2]. The 40S is shown in yellow, tRNA in brown, HCV IRES in green and blue (our structure) and orange (eIF5B-tRNA bound structure).

| **Supplementary Table 1. Data collection, model refinement and validation statistics of the 40S-HCV IRES complex** | |
|---|---|
| **Data collection** | |
| Particles | 404,357 |
| Pixel size (Å) | 1.39 |
| Defocus range (µm) | 1.5-3.4 |
| Voltage (kV) | 300 |
| Electron dose ($e^-/A^2$) | 20 |
| **Reciprocal space data** | |
| Spacegroup | P1 |
| *a*, *b*, *c* (Å) | 300.24, 300.24, 300.24 |
| α, β, γ (°) | 90, 90, 90 |
| **Refinement** | |
| Resolution range (Å) | 40.0-3.9 |
| Applied geometry weight (wxc) | 1.0 |
| No. reflections | 954,150 |
| R-factor | 0.233 |
| No. residues | |
| Protein | 4945 |
| RNA | 1922 |
| Ligands ($Mg^{2+}$ x $6H_2O/Mg^{2+}/Zn^{2+}$) | 24/74/3 |
| B-factors overall | 119.1 |
| Protein | 123.2 |
| RNA | 115.6 |
| Ligands ($Mg^{2+}$/coordinated $H_2O/Zn^{2+}$) | 39.4 |
| R.m.s. deviations | |
| Bond lengths (Å) | 0.008 |
| Bond angles (°) | 1.15 |
| **Validation** | |
| Protein | |
| Molprobity clashscore | 13.1 |
| Ramachandran plot | |
| Favored (%) | 95.6 |
| Allowed (%) | 4.2 |
| Outliers (%) | 0.2 |
| RNA | |
| Correct sugar puckers (%) | 94.4 |
| Backbone conformation outliers (%) | 0.0 |

**References**


1. Hashem Y, *et al.* Structure of the mammalian ribosomal 43S preinitiation complex bound to the scanning factor DHX29. *Cell* **153**, 1108-1119 (2013).

2. Yamamoto H, *et al.* Structure of the mammalian 80S initiation complex with initiation factor 5B on HCV-IRES RNA. *Nat Struct Mol Biol* **21**, 721-727 (2014).
